# The Impact of Acetic Acid on Measuring Ethanol Concentrations in Water and Human Serum Using Short-Wave Infrared Spectroscopy

**DOI:** 10.3390/ijms24032980

**Published:** 2023-02-03

**Authors:** Szymon Paprocki, Meha Qassem, Panicos A. Kyriacou

**Affiliations:** Research Centre for Biomedical Engineering, School of Science and Technology, University of London, Northampton Square, London EC1V 0HB, UK

**Keywords:** ethanol, acetic acid, short-wave infrared, spectroscopy, SWIR, human serum, water

## Abstract

Ethanol intoxication, although an elemental part of life in many places around the world, still presents several issues associated with excessive consumption. These issues range from drunk driving, violence, and antisocial behavior to self-harm, all exerting an increased cost on the society. Monitoring of intoxication levels can help to limit the impact of these issues by preventing the use of automobiles or heavy machinery and personal monitoring. Previous works on noninvasive measurement of ethanol tissue concentration for estimation of blood alcohol concentration (BAC) performed worst during the first hour of intoxication. Gas chromatography research of intoxication shows that levels of acetic acid rise together at a similar rate as those of ethanol after initial imbibement. In this research, short-wave infrared (SWIR) spectroscopy was utilized with the aim of establishing the interaction between ethanol and acetic acid in water and serum mixtures. The most consistent and clear correlation between ethanol and acetic acid was recorded at 2262 and 2302 nm wavelengths. Partial least-squares (PLS) analysis indicates that the most effective region for consideration in measurement of ethanol is the therapeutic window four (IV) due to high variance in vibration of carbon bonds. The behavior of spectra at different concentration ranges was examined and described in detail in relation to the consequence of alcohol measurement. The investigation concluded that ethanol shows distinctive regions of absorbance at wavelengths of 2262 and 2302 nm, with variations arising from increasing concentrations of acetic acid, whilst also showing that therapeutic window four is amongst the most influential regions of the spectrum for SWIR.

## 1. Introduction

Ethanol is amongst the most consumed and abused intoxicating substances in the world. According to the World Health Organization (WHO), alcohol consumption is a causal factor of over 200 diseases and 3 million deaths each year worldwide, representing 5.3% of all deaths [1]. As a consequence of its destruction and damage to society, several technologies and techniques have been developed over the last 100 years to quantify the intoxicating influence of ethanol and to reduce and police its consumption, especially in cases of driving under influence (DUI). Traditionally, the most used method of ethanol testing was through the use Breath Alcohol Concentration (BrAC) measuring devices. Although widely implemented, major shortcomings are associated with this type of measurement. These include errors associated with gender, alveolar lung volume, temperature, and the type of BrAC measuring device used [2,3,4,5,6,7]. Nonetheless, their ease of use, affordable cost, and portability have made these devices the primary tool for field intoxication monitoring.

Amongst other methods of measuring intoxication levels, much modern-day research aims to extract ethanol or ethanol-related metabolites from the surface of the skin [8,9]. These methods, although promising, are still in development stages, with only a handful deployed into the market. The gold-standard measurement for blood ethanol concentration is the use of headspace gas chromatography [10]. However, this method is very expensive and time-consuming, hence it is used sporadically. Reviews of ethanol and intoxication state devices and methodologies are summarized in many reviews, encompassing fields of chemistry, optics, and machine learning, to name a few [11,12,13,14].

In more recent years, increased interest towards personal monitoring through smart wearable devices has been aimed at helping to manage consumption and subsequent intoxication, mostly through implementations of novel tissue spectroscopic technologies to obtain spectral signatures of tissue, which when later analyzed can be used to determine analyte concentrations in the medium.

One technique that has been receiving particular interest over the last decade is tissue optical spectroscopy [15,16,17], especially the near-infrared/short-wave infrared (NIR/SWIR) range of the spectrum, spamming the wavelength ranges of 500–2600 nm to measure the vibrational mode changes associated with the presence of ethanol in blood and tissue, as well as breath. Some of these techniques have already found their implementation in real-life devices, with TruTouch TTT1100 Guardian & TTT2500 AlcoSense and Autoliv in-vehicle breath system [18,19]. The technology for noninvasive spectroscopic measurements using tissue and breath optical signatures is currently being implemented by the Driver Alcohol Detection System for Safety (DADDS) [7], with the first fleet of vehicles equipped with touch and breath sensors currently being tested in Florida, USA. The system will not allow an intoxicated driver to engage the vehicle if the detected alcohol level is above the driving limit.

The most prominent work in the subject of optical ethanol tissue sensing originates from publications associated with TruTouch Technologies (Riverside, CA, USA) concerning the optical behavior and modeling of interstitial fluid and tissue during intoxication and the development of TTT1100 (MARK1) and TTT2500 (MARK2) noninvasive ethanol sensing platforms, with the next iteration of the platform intended to find its way into commercial vehicles (MARK3). The performance of MARK2 was outlined in the works by Ridder et al. [20,21,22,23], showing clear superiority of the noninvasive system to a top-end laboratory-calibrated breathalyzer and a very close correlation between the gas chromatography reference of blood. However, several findings of this study present very pressing questions regarding the measurement of ethanol during the initial period of imbibement.

The results from Ridder et al. show a significant difference between the results from the sets “All Data” and “Elimination only”, which presents a question about the performance of the system during the “Absorption” stage. This data was not presented by the authors separately, but instead were combined into the “All Data” data set. After analysis of the published figures, it was discovered that the correlations between the optical measurements from the finger and the forearm during the first hour of the study were far more erroneous that those collected after the intoxication peak. This effect was particularly prominent in measurement from the forearm, changing the correlation from 0.54 for “All Data” to 0.88 for the “Elimination only” stage, an increase in correlation of 0.34 and a decrease in root-mean-squared error (RMSE) of 16.2 mg/dL [20].

**Figure 1:**
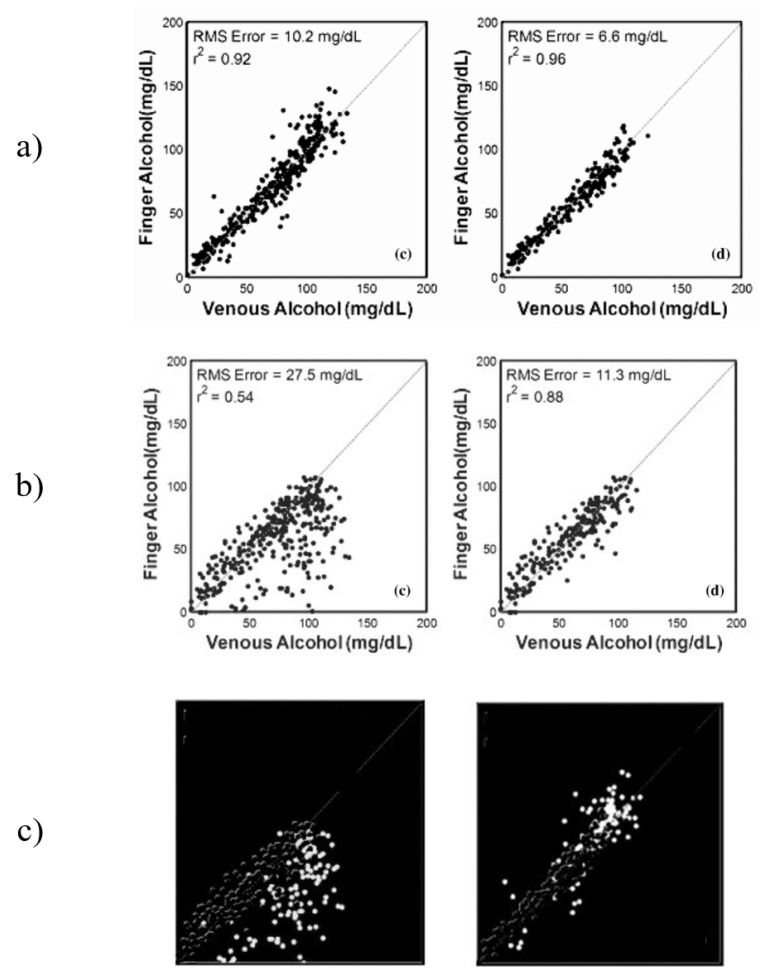
Part of the results obtained by Ridder et al from their in vivo study. (**a**) finger data; (**b**) forearm data [20]; (**c**) results from the first hour, forearm and finger from left to right.

Previous work on noninvasive spectroscopic sensing of ethanol intoxication conducted by TruTouch presented a comparison study between spectra responses obtained from the finger and forearm under the influence of ethanol [20]. The study used gas chromatography as a primary reference and a laboratory-calibrated breath alcohol device. The obtained results from the in vivo trial are presented in Figure 1a–c, respectively. Figure 1a (finger) presents results for “All Data”data and the “Elimination only”stage (left to right). The “Elimination only” showed a slightly improved predictive ability and lowered the RMSE by a factor of almost two. A very similar increase in predictability can be seen from Figure 1b (forearm), but with a much greater impact. The “Elimination only” stage achieved an almost 2.5 lower RMSE as compared to “All Data”. This presents a very interesting case of analyte detection, where the elimination of data points from a particular period of intoxication improves the results by a significant factor. The authors of this research attribute the difference in predictive ability to two factors: probe design and skin thickness. However, both factors are inconsistent with the methodology and the results of the study. The methodology included design of two probes, each one specifically designed to maximize data collection from the selected site. If the differences between the two data sets are to be contributed to the difference in probe design, the optimization of the design to maximize data collection from each site would have not been considered. However, each of the probes designed in reflectance mode considered the surface of the measuring site, such that in the case of the forearm sensing probe includes a 25-degree separation angle to maximize photon collection. This would explain the general difference between the data collected from the finger as compared to the forearm; however, this difference would remain near-constant in all stages of the experiment. The skin thickness of the two regions remains the same throughout the entire investigation, hence additional factors may have contributed to the difference between the data sets that resulted in the changes observed between the time sets of the investigation, such as the presence of an interferent analyte not accounted for in the calibration sets. Figure 1c presents the removed data points between the “All Data” and “Elimination only” sets for forearm and finger, respectively. The difference in performance can be clearly seen, especially in the forearm data with almost all data points being below the line of best fit. A similar case can be seen with the finger data, but to a much lower effect.

**Figure 2:**
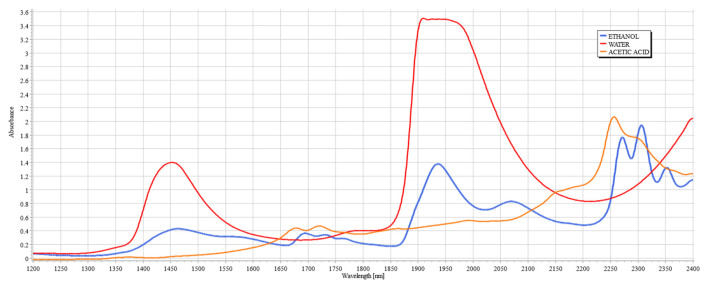
Spectra of water (red), ethanol (blue), and acetic acid (yellow) between 1200 and 2400 nm.

The metabolism of alcohol is a generally very well-understood topic in terms of the compounds produced during the process, including compounds such as acetaldehyde and acetic acid. These three compounds are the most prominent markers of alcohol metabolism facilitated by the enzymes alcohol dehydrogenases (ADH) [24]. Studies of blood chemistry during an intoxication period have been conducted to establish the correlation between their respective concentrations and classification of user type, such as alcoholics, based on the concentration of acetaldehyde and acetic acid with respect to ethanol. The results obtained from headspace gas chromatography of intoxicated blood by Tsukamato et al. [25] and Giles et al. [26] show that the concentration of acetic acid rises rapidly during the first hour from initial consumption. Acetic acid then remains at the peak level for a period of 2–3 h before decreasing to lower concentration levels, yet remaining at elevated concentration from its original level for up to eight hours. Hence, the increased presence of acetic acid in the blood during the absorption period may be a contributing factor to the decreased accuracy of the developed models in previous publications. This becomes more apparent when the spectroscopic signatures of ethanol and acetic acid are compared in the SWIR range, as shown in Figure 2.

Within the range of SWIR spectroscopy (1100 to 2600 nm), four regions are of interest due to the high variability of vibrational modes of organic compounds and deep light penetration. These regions are designated as “therapeutic windows” and are numbered using Roman numerals (I, II, III, and IV). The location of the therapeutic windows are I: 650–950 nm, II: 1100–1350 nm, III: 1600–1870 nm, and IV: 2100–2300 nm. These bands are particularly useful in measuring tissue composition and property due to deep penetration. In the regions outside of these windows, the penetration depth is shortened due to the presence of water bands, which have high absorbance outside of therapeutic windows [27].

**Figure 3:**
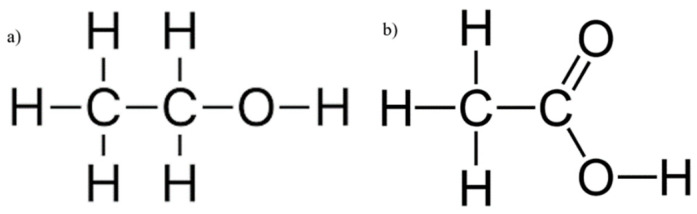
Chemical structures of ethanol and acetic acid, respectively.

Figure 2 shows the spectral signatures of ethanol and acetic acid alongside water, clearly illustrating the justification for the existence of the therapeutic windows, since they avoid the peaks of water, a major component of interference in the measurements of analytes of tissue due to its high concentration in tissue. Figure 3a,b illustrate the skeletal structures of ethanol and acetic acid, respectively. Since acetic acid is a by-product of ethanol metabolism by ADH [24], it shares many of its structural components, such as the length of the carbon chain and the O–H bond, but it is particularly distinctive from ethanol by the presence of C=O stretching and bending. As both compounds increase in concentration during an intoxication episode, their interference in the SWIR spectrum will be evident. The following study aims to establish the extent of this influence, imitating the conditions of intoxication.

When considering the use of SWIR to measure the concentrations of compounds such as ethanol and acetic acid, the therapeutic windows that seem appropriate for consideration are windows II, III, and IV due to the clear breakout of peaks above water, hence allowing for clear measurement from a known baseline. Previous works from TruTouch Technologies considered only two windows: III and IV, located on either side of the major water band [21]. It is key to consider that window IV is a region of higher water absorbance than other therapeutic windows, yet the scattering properties at that region are generally classified as minimal, and hence the balance between absorption and scattering in that region must be carefully considered when examining tissue composition. It is also a region where ethanol and acetic acid combinational bands show very strong absorbance, thereby making possible the quantification of these compounds.

As personal monitoring is slowly becoming a norm in smart-wearable devices, systems capable of accurately monitoring intoxication levels at all stages of an intoxication episode are of particular value, as they would allow for accurate monitoring of intoxication regardless of the frequency of alcohol consumption, and even inform the user if their drinking habits are becoming dangerous. The following study aims at quantifying the variance in absorbance levels of ethanol peaks of 2262 and 2302 nm with respect to the natural variance of acetic acid at those peaks. Further PLS analysis aimed to establish the most effective window for ethanol level prediction.

## 2. Results and Discussion

**Figure 4:**
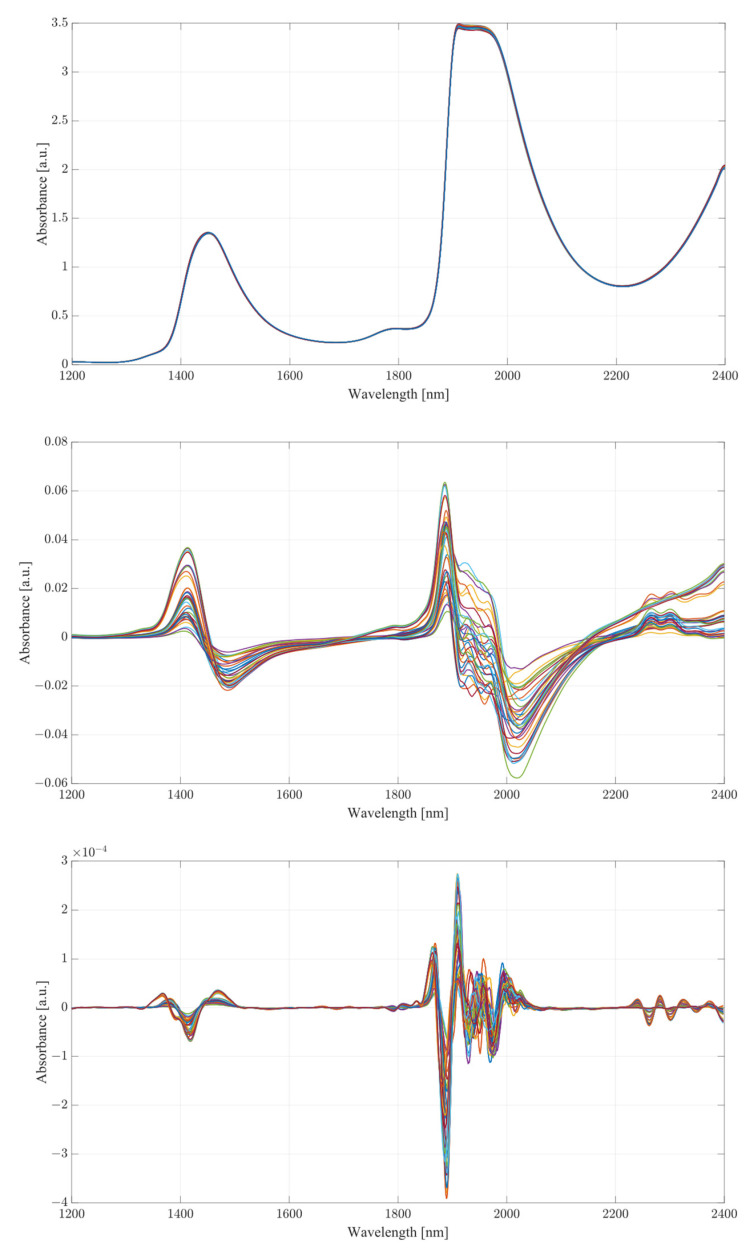

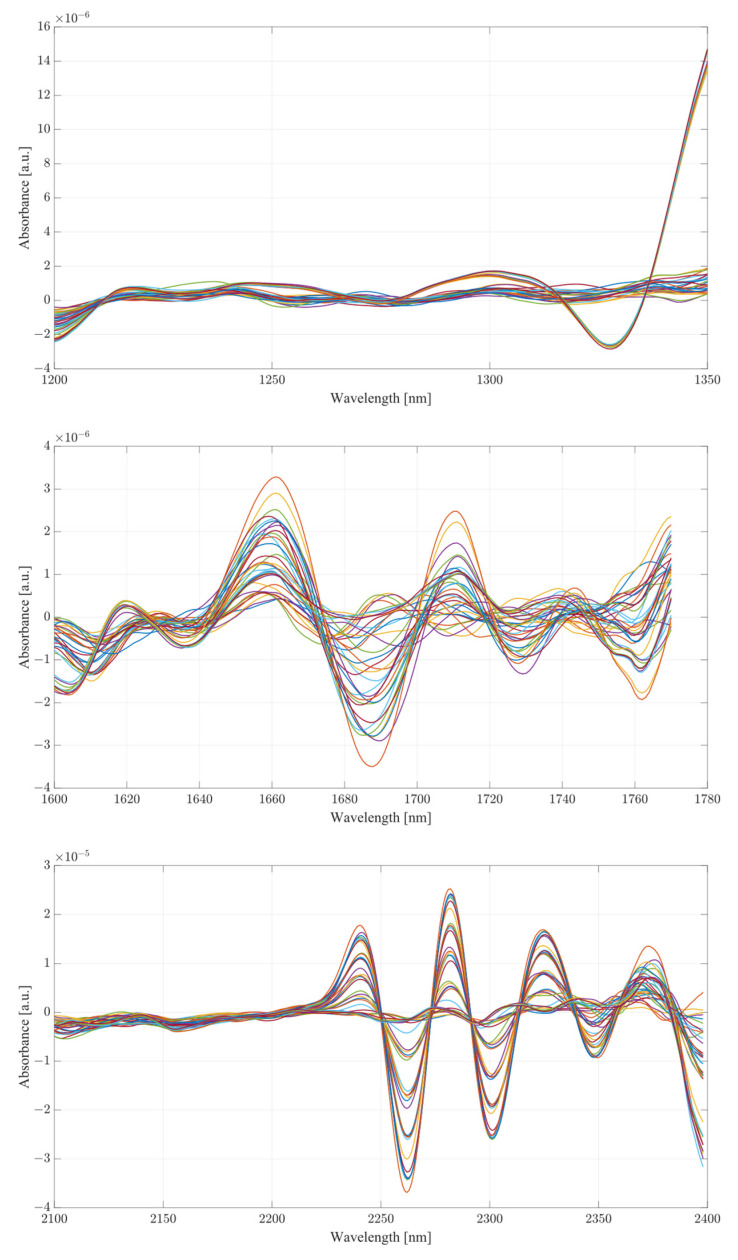
Water mixture results, from top to bottom: raw spectra, postbaseline subtraction, second derivative, therapeutic window II, therapeutic window III, therapeutic window IV.

**Figure 5:**
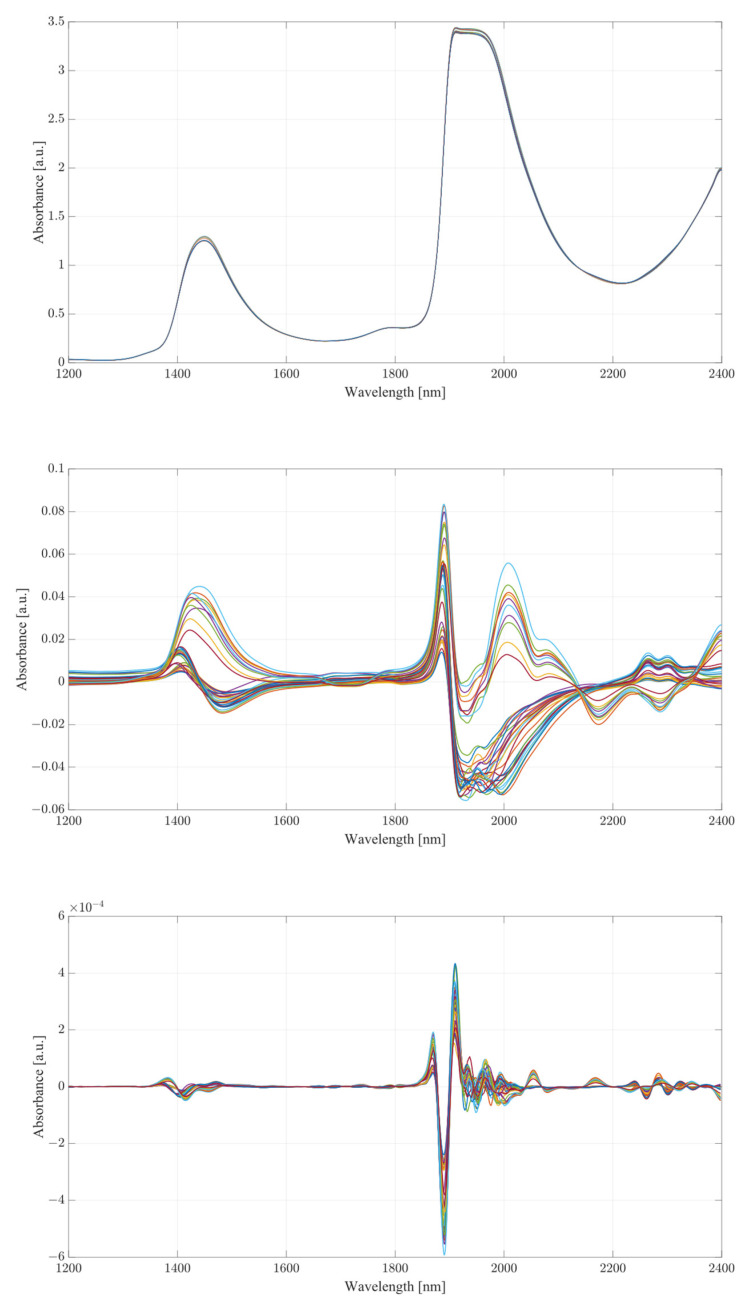

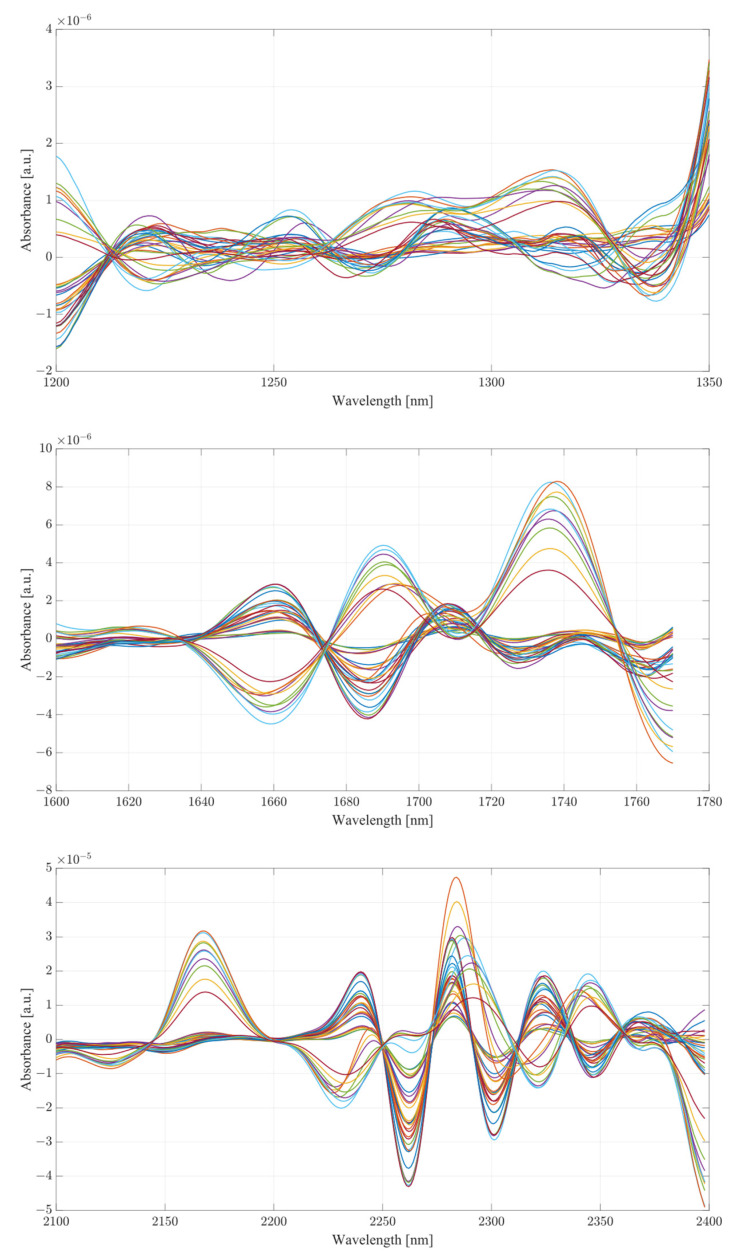
Serum mixture results, from top to bottom: raw spectra, postbaseline subtraction, second derivative, therapeutic window II, therapeutic window III, therapeutic window IV.

The obtained spectra from water and serum mixtures are displayed in Figure 4 and Figure 5, respectively. The spectra obtained were preprocessed by removing the baseline of the mixture (water or serum), then smoothed and differentiated (2nd). The obtained results show changes across almost the entirety of the acquired spectral region, with the most prominent changes occurring at the water bands and the therapeutic windows. Previous works on the subject of NIR/SWIR investigation of water-based mixtures showed that strong hydrogen bond interactions are present and are responsible for many of the influences on the spectrum [28,29].

The spectra acquired from mixtures in water, in the case of raw spectra on their own, show no variation between samples, and appear as a single bold line. Once the baseline of deionized water was subtracted, greater variation was revealed. The regions where the greatest variation in sample absorbance occurred were between 1350 and 1600 nm, suggesting a strong influence of ethanol and acetic acid on water absorbance bands due to the O-H bonds on both compounds. This is further reinforced when a region between 1850 to 2100 nm is considered, showing clear distinction across all samples. Generally, it is unwise to consider these regions in measurements of analyte concentrations in water-based mixtures due to the very high absorbance of water and high levels of noise arising from scattering from O-H water bond stretching, particularly in the 1850 to 2100 nm region. The region between 2100 and 2400 nm, however, provides further information about the behavior of those mixtures. Most of the variation across samples was concentrated between 2250 and 2350 nm, a region where ethanol absorbance was higher than that of water; however, an observation about the results in the region shows that the behavior of water/ethanol and water/acetic acid mixtures was not the same as that of all three mixed together. Samples containing only one analyte show higher absorbance levels than those of mixtures. This suggests an interaction between the two analytes in the mixture itself, resulting in lowered absorbance across the region. This difference is likely the result of the interaction of O-H bonds of ethanol and acetic acid and the C=H bond of acetic acid. Regions of windows II and III show moderate variation between samples, with single-analyte samples distinguishing themselves from the remainder of mixtures [30].

The second derivative spectra provide additional insight into the changes in absorbance between levels of acetic acid and ethanol in water mixtures. The regions of most apparent differences in absorbance included both water bands at 1350 to 1600 nm and 1870 to 2100 nm. The combinational band region between 2100 and 2400 nm shows strong variation between samples. Therapeutic region II shows less significant variation between samples, with pure samples exhibiting a different behavior from all the rest of the mixtures. Pure samples had an increased absorbance from around 1330 nm, whilst all the mixture absorbances remained at low values around the zero point. Therapeutic window III shows a much greater deal of variation, with distinctive regions of changing absorbance at 1660, 1685 and 1710 nm. Window IV provides the clearest picture about the behavior of acetic acid and ethanol mixtures. The region between 2100 and 2220 nm was mostly uneventful when compared to the absorbance variation between 2220 and 2400 nm. The variations between samples are clearly noticeable, but the variations also form a region of high concentration separated by gaps, forming five distinctive regions, corresponding to the levels of ethanol. Acetic acid influence can be seen from the slight variations across each of the five levels.

Considering the spectra acquired from serum mixtures compared to those of water, human serum demonstrated clear differences from the initial loading of the data, seen in the small variation of raw spectra in the absorbance at the water peaks. Once the baseline of the serum was subtracted, the regions of variation between samples was clearly visible. Water absorbance bands, similarly to deionized water, were strongly influenced by both ethanol and acetic acid. However, a clear distinctive pattern occurred with samples only containing a single analyte. The differences between mixture samples and single-analyte samples can be seen from subtracted baseline regions 1350 to 1600 and 1880 to 2400 nm. The differences between the pure and sample mixtures follow those seen in single-analyte samples in water. Whilst separated, ethanol and acetic acid produce similar responses between each other; when in combination, they form a different morphology altogether. In terms of the differences amongst pure samples of ethanol and acetic acid, the general shape of the ethanol absorbance region remained mostly unchanged with the distinctive absorbance region of combinational bands, yet not as pronounced when coupled with acetic acid. The combination of the two compounds in human serum suggest an active interaction between them. Therapeutic windows II and III show a similar level of variance across the range similarly to the water mixtures. 

The second derivative spectra show clear variations across all therapeutic windows, to a much greater degree than those seen in water mixtures. This may be due to the presence of other organic molecules in the serum, which contribute to the increase in absorbance across all regions. Therapeutic window IV, similarly to water mixtures, shows a clear large separation between concentrations of ethanol, with smaller variance between each ethanol level depending on the concentration of acetic acid. Similarly to water, the second derivative of therapeutic window IV shows a clear correlation at wavelengths of 2262 and 2302 nm.

Both sample sets show that when in combination, ethanol and acetic acid form regions of regular change in absorbance depending on concentration levels. To examine the variance between the spectra whilst considering both ethanol and acetic acid, the most promising correlation wavelengths of 2262 and 2302 nm were examined in terms of absorbance change with respect to acetic acid concentration for both water and serum mixtures. Analysis of the influence of acetic acid was quantified in Table 1. The key metric in evaluating the influence of acetic acid on the ethanol peaks of 2262 and 2302 nm was the ratio of the variance, calculated using the variance occurring between the spectra of acetic acid and water/serum mixtures. By considering the variance ratio between the mixture samples and only acetic acid samples, the changes in the ethanol peaks’ absorbance were quantified with respect to the natural variance of acetic acid absorbance.

**Figure 6:**
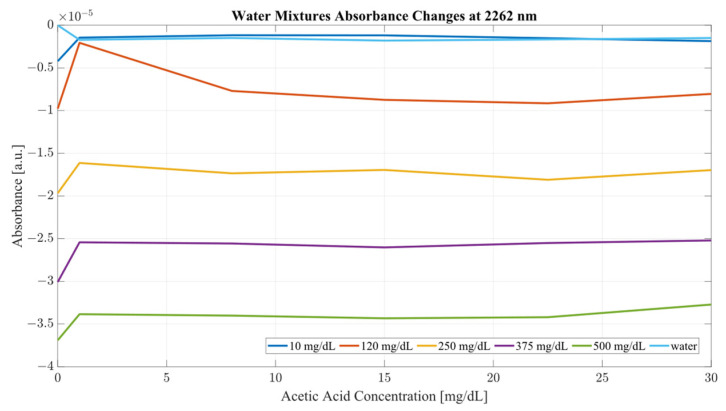
Water mixture absorbance changes at 2262 nm.

**Figure 7:**
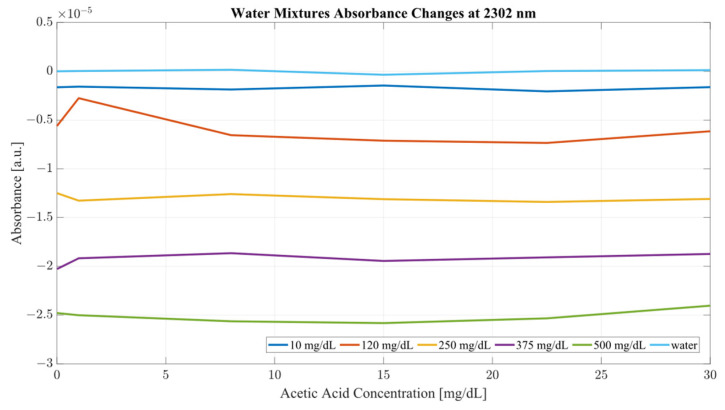
Water mixture absorbance changes at 2302 nm.

**Figure 8:**
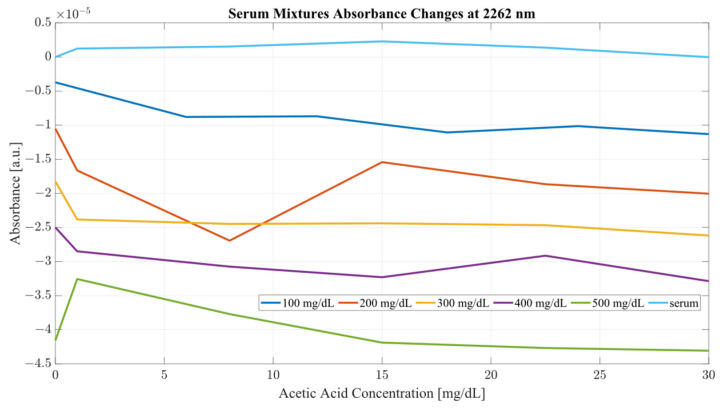
Serum mixture absorbance changes at 2262 nm.

**Figure 9:**
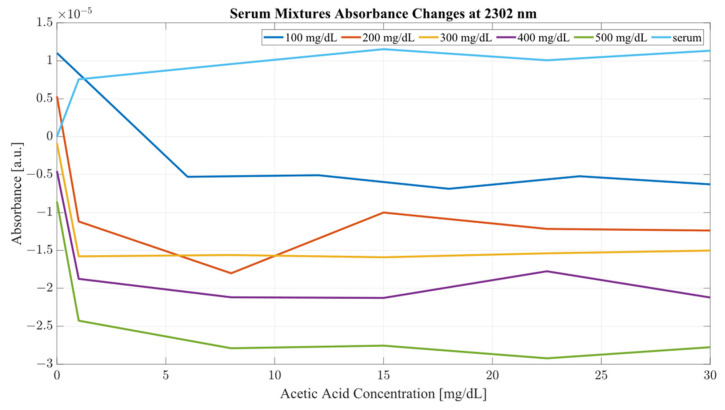
Serum mixture absorbance changes at 2302 nm.

A closer analysis of the absorbance changes between the levels of acetic acid at five different levels of ethanol are shown in Figure 6, Figure 7, Figure 8 and Figure 9, where both data sets demonstrate the greatest variation in absorbance as resulting from the concentration of ethanol in each of the mixtures. The influence of absorbance change is greater in water than in serum when comparing the plot line for 500 mg/dL samples from both data sets as well as the ratio of their variance. In the case of water, the variance ratio reached 4.18 and 12.73 as compared to serum, with 19.91 and 3.28 for 2262 and 2302 nm, respectively. Across both data sets and each ethanol concentration, the influence of acetic acid can be seen to some degree between concentrations, but not near the influence seen from ethanol. This is likely due to the difference in the concentration of acetic acid and ethanol order of magnitude. The concentration of ethanol was varied over a much larger scale than those of acetic acid, explaining the limited variation. Nonetheless, the changes occurring due to the presence of acetic acid increased the absorbance at all concentrations of ethanol, with some sample sets of ethanol concentration showing a very large alteration in magnitude and area than others. This is clear for ethanol and water at 2262 nm for samples of 10 and 250 mg/dL, with 120 mg/dL showing large variation (17.23 at 2262 and 85.06 at 2302 nm). This type of interaction could be indicative of increased interference of combinational bands at higher concentrations, resulting in greater variation in absorbance across this concentration. This influence in variation is also seen in the serum samples, but not to the same degree as in the water, most likely due to the presence of other organic molecules further interacting with the analytes.

The impact of acetic acid on the absorbance levels of distinctive ethanol bands is more clearly defined at 2302 nm. As seen in Figure 7, increases in acetic acid result in irregular changes in absorbance levels for all levels of ethanol. In the case of all levels of acetic acid, the original absorbance of ethanol specific peaks changed in absorbance by a significant margin. As seen in Figure 8, the levels of distinctive ethanol bands change between the original absorbance of ethanol and after the addition of acetic acid. This fluctuation is not strictly limited to the peaks of 2262 and 2302 nm, but occurs throughout all therapeutic windows. Peaks of 2262 and 2302 nm, however, show a clear distinction between ethanol levels; however, this difference is altered at different levels of acetic acid. 

Furthermore, the same analysis was performed on human serum, yielding Figure 8 and Figure 9. The results from peak absorbance analysis of wavelengths 2262 and 2302 nm show that the absorbance changes associated with increased concentration of acetic acid are very small in magnitude in water samples. Water mixtures show a slight level of fluctuation from their original absorbance, suggesting an interaction of C–C bonds in the mixture. However, considering serum mixtures, the absorbance levels at 2262 and 2302 nm show a far greater variation. Across all concentrations of acetic acid, both wavelengths show high levels of variation from their original absorbance. Moreover, the change in absorbance across all ethanol concentrations show a similar change in intensity after the addition of acetic acid into the mixtures. This is indicative of the impact of other organic molecules in the serum, which, when acetic acid is present, react and produce a high level of variation in absorbance as the concentration of the acid is increased. 

It is key to consider that although acetic acid does not have a distinctive peak at 2262 or 2302 nm, its presence results in variations in absorbance for all levels of ethanol, particularly pronounced in the results from the human serum. The absorbance region of acetic acid occupies the same regions as ethanol, as seen from Figure 2. Another important consideration is the proportion of the two compounds with each other. The concentration range for ethanol was 500 mg/dL, whilst the acetic acid range was only 30 mg/dL. With that proportion, it can be said that most of the absorbance change at those wavelengths is due to the increase in ethanol concentration. However, as seen from Figure 8 and Figure 9, the presence of acetic acid results in an increase in absorbance with the overlapping concentration of ethanol after the addition of acetic acid. This can potentially lead to an error in measurement when the acetic acids rise rapidly in the blood stream and water-rich layers of tissue, resulting in an inaccurate estimation of ethanol because of the presence of acetic acid.

However, concerning the measurement of ethanol, acetic acid interference presents a particular challenge for the distinctive wavelength estimation of ethanol concentration. To establish the optimal regions of measurement for the quantification of ethanol in the presence of acetic acid, several PLS models were constructed to establish the correlation of ethanol to the principal components of the recorded spectra. Seven PLS models were constructed for each mixture, f in total. The combination sets of the considered data are outlined in Table 2, together with their respective performance metrics.

As seen from Table 2, the differences in the predictive ability of PLS models varies greatly depending on the medium investigated. In the case of water mixtures, PLS models performed very well considering only therapeutic window II, and when any combination except for windows II and III were supplied to the model, the first latent variable (LV) accounted for at least 94.91 of the variances in the data sets. It can be said with certainty that most of the variance for in the PLS model of water is accounted for by the concentration of ethanol, suggesting a linear-like correlation between absorbance changes and the concentration of ethanol, proven by the plots of absorbance changes in Figure 5 and Figure 6. The second LVs of the PLS models in water mixtures accounted for between 0.77 and 24.79 percent of the variance; a large variance considering the changes in the concentrations of both compounds is systemic, clearly illustrating the difference in the influence of acetic acid in each of the therapeutic windows. Hence, it is key to consider the selection of the investigative region when measuring concentrations of a volatile organic compounds such as ethanol and acetic acid. The greatest RMSE occurred for therapeutic window II, at 158.24 mg/dL, in contrast to the LV1 value, suggesting a very low level of variation between samples. The best performance in terms of RMSE was achieved by therapeutic window III, with only 18.36 mg/dL, followed closely by therapeutic window IV, at only 22.35 mg/dL. In separation, these two windows provided a relatively good regression model; however, their combination achieved 24.34 mg/dL RMSE, the third lowest in the set. Models including window IV also show relatively low levels of RMSE, varying between 24.34 and 26.90 mg/dL. When all three therapeutic windows were considered, the RMSE only reached 25.73 mg/dL, suggesting that perhaps using only two of the three regions will yield better results than the use of all three together.

The PLS models of serum mixtures present a very similar, yet distinctively different pattern in the explained variance and RMSE. Generally, models only considering a single therapeutic window show very low results of the first LV, and an almost equal result for LV two, which is significantly different from the results obtained in the same therapeutic windows for the water mixtures. Windows II and III also show very high RMSE, indicating low recognition between samples. In the case of single therapeutic window models, the best performance was achieved by window IV, with almost half the error of the other two windows, and a higher principal component one. Combinational models performed significantly better than in separation, achieving an average principal component one score of 72.92, much lower than those seen in the water mixtures. The lowest RMSE was achieved by combinations of II and IV as well as III and IV, at only 30 mg/dL each, whilst windows II and III reached an RMSE of 90.61 mg/dL, triple that of the other two combinations. The model using all three windows performed relatively worse than its counterpart in the water mixtures, achieving more than double the error seen from combinations using therapeutic window IV. It is important to note that for all the models obtained from the serum samples, the first LV accounted for an average of 60.68% of the variance, whilst the second component accounted for an average of 33.3% of the variance. This suggests a very different pattern of behavior in terms of variance explained as compared to water mixtures. Serum mixtures are much more complex than pure water, hence the presence of other organic molecules may impact the vibrational modes of the mixture. It also important to consider that the interactions of the vibrational bonds in serum mixtures are much more prevalent than those of water. Greater number of C–C and C=O bonds results in a variety of morphological changes in the spectral regions of the therapeutic windows.

**Figure 10:**
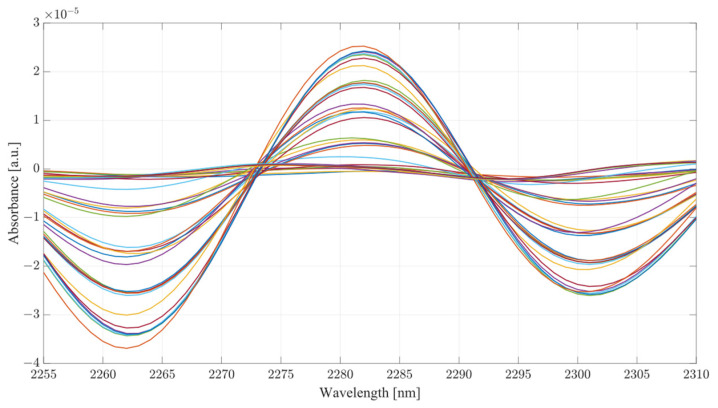
Peaks of 2262 and 2302 nm in water mixtures.

**Figure 11:**
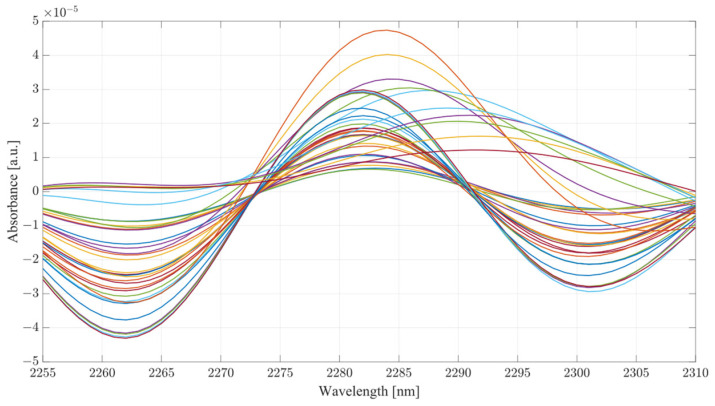
Peaks of 2262 and 2302 nm in serum mixtures.

Considering the analysis of the data obtained during this investigation, several conclusions can be drawn from the results. From the PLS analysis of the therapeutic windows, water mixtures showed very little variation with respect to the increasing concentration of acetic acid, suggesting that the presence of the acid is largely unnoticeable in water mixtures. However, in the case of the serum mixtures, the presence of acetic acid results in a substantial change in the absorbance, so much so that the first LV was not able to account for more than 78 percent of the variance. The second component also carried a significant influence on the PLS results, accounting for at least 19 percent of the variance. PLS models are heavily influenced by the data set supplied to them; however, this is provided that the data fed into the model contain enough variation to limit the RMSE. It is the opinion of the authors that the most significant region for measuring ethanol concentration using SWIR is therapeutic window IV. Window IV has presented very good results, both in water mixtures and serum. The PLS analysis shows that models including window IV outperformed all other models. This gives a clear indication that the region where concentrations of ethanol are most distinctive lies within window IV, with windows II or III acting as a supporting element in the determination of concentration, not as the major distinctive region. Therapeutic window IV is particularly unique as it represents the vibrational modes of the C-C bonds, which are present in mixtures such as serum. Hence, consideration of the change of a compound concentration, which has two distinctive wavelengths of absorbance in that region, is strongly correlated. It is also the region where the normal baseline absorbance would be largely unaffected, hence the measuring of change in that region proves particularly useful. This becomes clear when the second derivatives of TW IV at wavelengths of 2262 and 2302 nm are considered, as seen from Figure 10 and Figure 11 for water and serum, respectively. The changes in absorbance occurring at peaks of 2262 and 2302 nm are closely correlated with the changes in ethanol concentration for both serum and water; however, the addition of acetic acid into the mixtures causes fluctuations in absorbance magnitude at every ethanol level. Figure 11 provides a more detailed picture for the behavior of ethanol/acetic acid mixtures in serum. Primarily, the difference between the two spectra is the fact that serum mixtures present a far less distinctive pattern for ethanol level, hence making it more difficult to establish the ethanol concentration in the mixture. Key to note also is that serum mixtures with only ethanol and acetic acid presented different morphological behavior when compared to the more complex mixtures. Peaks of 2262 and 2302 nm were chosen for this investigation because they showed a strong correlation between absorbance changes and ethanol concentration, as well as being located within TW IV. The rotations responsible for these peaks are thought be originating from CH stretch/bend combinations [31].

## 3. Materials and Methods

The following methodology was used to establish the influence of ethanol and acetic acid on water and human serum. The collected spectra occupied the SWIR region, and the concentrations of ethanol and acetic acid were varied across a full range of concentrations, including the extremes. The collected spectra were then preprocessed and analyzed for correlation between acetic acid and ethanol at wavelengths of 2262 and 2302 nm. PLS models were later run on individual therapeutic windows and their combination to establish the most influential region of performance.

### 3.1. Sample Preparation

To examine the impact of acetic acid on the absorbance bands of ethanol, two sets of mixtures were prepared. Both sets consisted of 36 samples, with ranging concentrations for ethanol and acetic acid between 0:500 and 0:30 mg/dL, respectively. The samples consisted of 10 samples of medium and single analyte only, 25 samples of mixtures of both analytes at the 6 different levels, and 1 baseline spectrum of serum and of water. The samples were prepared using deionized water (Water Company, London, UK) and human serum (mixed pool, sterile filtered 0.2 uL, off the clot (TCS Biosciences LTD, Buckingham, UK)). This specific type of serum was chosen due to the lack of coagulants, which themselves contain O-H bonds and could potentially interfere with SWIR measurements. The same type of serum was used in previous publications concerning analyte detection in bodily fluids, specifically lactate [16]. All the samples were prepared at 24.2 degrees Celsius. Each set of results was obtained over a period of two days. In the case of serum, all the data were acquired within 48 h of being delivered to ensure its biological viability. The serum was placed in a fridge at 5 degrees Celsius when not in use. Each sample of the mixture was prepared in 2 mL cap-sealed tubes and stored in the fridge when not in use.

### 3.2. Spectroscopy

Three consecutive absorbance spectra were collected between 1200–2400 nm for each sample. Each sample from each set was scanned in a random order. A total of 216 spectra were acquired. The spectrophotometer used for spectra acquisition was the Lambda 1050 dual-beam spectrophotometer (Perkin Elmer Corp, Waltham, MA, USA). The resolution of the collected spectra was kept at a step interval of 1 nm. All sample spectra were averaged and used for analysis. A halogen tungsten lamp was used as a light source and polycrystalline lead sulfide (PbS) was used as a detector. The response time was kept at 0.2 s. No attenuation was applied to the reference beam. 

The baseline correction of 100% transmittance/0% absorption was also introduced in the spectrophotometer to remove any effect of ambient environment on the spectra; 1 mm quartz cuvettes (Hellma GmbH & Co., Mulheim, Germany)) were used to introduce the samples into the spectrophotometer. The reference cuvette was always kept empty.

### 3.3. Spectra Analysis

The obtained spectra were displayed, processed, and organized in MATLAB™ R2022b (MathWorks, Natick, MA, USA). All the spectra were pretreated using baseline removal, smoothing (SG) (order = 3, window size = 25), and mean center. Analysis was performed on second derivative spectra due to the enhanced visual resolution and to suppress constant background interference. An important consideration for processing and analyzing derivative data is the transfer of maxima peak into minima peaks and vice versa. The obtained results were analyzed for correlation trends between concentrations. PLS analyses were applied to each of the three therapeutic windows and all their combinations. Cross-validation was carried out using venetian blinds (4 data splits, blind thickness = 2).

## 4. Conclusions

Given that the most effective region for measuring ethanol concentration is therapeutic window IV, is also clear that the most important region in that window are the peaks of ethanol, at 2262 and 2302 nm. The analysis of each of these wavelengths in terms of their absorbance change with respect to acetic acid showed a clear disturbance from the original absorbance of the ethanol samples. The impact of acetic acid on ethanol absorbance was largely limited in the case of water mixtures. Clear distinctive regions of ethanol levels were recorded. However, increasing the concentration of acetic acid did not influence the general absorbance by a large degree; only the initial addition of the acid resulted in irregular changes in ethanol absorbance. This was the case for both wavelengths 2262 and 2302 nm. The same analysis of the serum samples showed a far greater change at all levels of acetic acid. The changes seen from the addition of acetic acid resulted in a steep drop in the absorbance, in many cases so significant that it resulted in an overlap of concentrations, indicating a difference of at least 150 mg/dL from the original position of the baseline ethanol level. The most significant impact of acetic acid can, however, be seen in the peak of 2302 nm, where a small concentration of acetic acid (6 mg/dL) resulted in large changes in the absorbance. Therefore, in efforts to establish the impact of acetic acid on the ethanol absorbance bands, the work presented a study of the influence of acetic acid on the morphology of the ethanol spectra in water and human serum along the concentrations aiming to represent intoxicating conditions. An important consideration in this study were the ranges of the compounds used: ethanol and acetic acid. Naturally, acetic acid is present in the blood stream at very low quantities, whilst ethanol is almost nonexistent. However, during an intoxication period, the level of ethanol increases, and so does the level of acetic acid. During such episodes, it can be accounted that for every 80 mg/dL of ethanol, 5 mg/dL of acetic acid is synthesized into the bloodstream. Typically, the maximum intake of alcohol resulting in death is around 350 mg/dL, hence using concentrations above that level is not truly representative of a regular intoxication period, but rather a serious addiction problem. Studies concerning levels of acetic acid at those intoxication levels are scarce and typically do not use a reliable reference, such as headspace gas chromatography. It is possible that the concentration ranges of acetic acid in this study are not fully representative of those found during an intoxication episode. However, they provide an insight into the extent of the variation associated with their concentration on the spectra, with a possible future application for monitoring for alcohol ketoacidosis [32]. Moreover, pilot studies on mouse brains have shown that acetic acid can affect the “reward system” of the brain in a similar way that ethanol does [33].

In conclusion, the presence of acetic acid in human serum shows significant changes in therapeutic window IV, which can be attributed to the lowered accuracy of the results obtained by Ridder et al. during the first hour of their in vivo trials. Models used for the prediction of ethanol levels using SWIR spectroscopy should mostly consider using therapeutic window IV as the main site of analyte detection, recognition, and measurement. Future investigations into noninvasive measurements of ethanol, especially in the first hour of intoxication, should consider the presence of by-products of ethanol, particularly acetic acid. Currently, the field of noninvasive spectroscopic sensing is limited by the technology costs and material requirements for production of emitters and detectors capable of capturing the changes in tissue absorbance associated with intoxication.

## Figures and Tables

**Table 1:** Statistical analysis of the absorbance variance.

Medium	λ	EtOH	Mean	Median	Standard Deviation	Variance	Variance Ratio
**Water**	2262	10	−1.89 × 10^−6^	−1.48 × 10^−6^	1.16 × 10^−6^	1.35 × 10^−12^	2.96
		120	−7.57 × 10^−6^	−8.39 × 10^−6^	2.80 × 10^−6^	7.86 × 10^−12^	17.23
		250	−1.75 × 10^−5^	−1.72 × 10^−5^	1.23 × 10^−6^	1.51 × 10^−12^	3.32
		375	−2.63 × 10^−5^	−2.55 × 10^−5^	1.87 × 10^−6^	3.49 × 10^−12^	7.65
		500	−3.43 × 10^−5^	−3.41 × 10^−5^	1.38 × 10^−6^	1.91 × 10^−12^	4.18
	2302	10	−1.70 × 10^−6^	−1.63 × 10^−6^	2.19 × 10^−7^	4.80 × 10^−14^	1.45
		120	−5.92 × 10^−6^	−6.35 × 10^−6^	1.67 × 10^−6^	2.80 × 10^−12^	85.06
		250	−1.30 × 10^−5^	−1.31 × 10^−5^	3.67 ×10^−7^	1.34 × 10^−13^	4.08
		375	−1.92 × 10^−5^	−1.91 × 10^−5^	5.91 × 10^−7^	3.50 × 10^−13^	10.62
		500	−2.51 × 10^−5^	−2.52 × 10^−5^	6.48 × 10^−7^	4.20 × 10^−13^	12.73
**Serum**	2262	100	−8.95 × 10^−6^	−9.46 × 10^−6^	2.79 × 10^−6^	7.79 × 10^−12^	9.31
		200	−1.80 × 10^−5^	−1.76 × 10^−5^	5.46 × 10^−6^	2.98 × 10^−11^	35.65
		300	−2.36 × 10^−5^	−2.44 × 10^−5^	2.75 × 10^−6^	7.57 × 10^−12^	9.06
		400	−2.98 × 10^−5^	−2.99 × 10^−5^	2.89 × 10^−6^	8.34 × 10^−12^	9.98
		500	−3.99 × 10^−5^	−4.17 × 10^−5^	4.08 × 10^−6^	1.66 × 10^−11^	19.91
	2302	100	−2.96 × 10^−6^	−5.26 × 10^−6^	6.89 × 10^−6^	4.74 × 10^−11^	2.53
		200	−9.74 × 10^−6^	−1.17 × 10^−5^	7.87 × 10^−6^	6.20 × 10^−11^	3.30
		300	−1.31 × 10^−5^	−1.55 × 10^−5^	6.00 × 10^−6^	3.60 × 10^−11^	1.92
		400	−1.75 × 10^−5^	−2.00 × 10^−5^	6.50 × 10^−6^	4.22 × 10^−11^	2.25
		500	−2.42 × 10^−5^	−2.77 × 10^−5^	7.85 × 10^−6^	6.16 × 10^−11^	3.28

**Table 2:** PLS models’ results.

	Model	LV1	LV2	R^2^	RMSE mg/dL
**Water**	II	98.73	0.77	0.26754	158.24
	III	82.93	9.15	0.99013	18.36
	IV	72.38	24.79	0.98539	22.35
	II and III	68.10	19.78	0.83976	74.01
	II and IV	97.01	2.45	0.97884	26.90
	III and IV	96.96	2.55	0.98267	24.34
	II and III and IV	94.91	4.07	0.98063	25.73
**Serum**	II	40.85	42.25	0.54499	115.20
	III	33.72	51.67	0.64592	101.64
	IV	52.58	44.16	0.89908	54.26
	II and III	78.84	19.84	0.71851	90.61
	II and IV	74.09	24.80	0.96820	30.46
	III and IV	74.42	24.50	0.96832	30.40
	II and III and IV	70.25	26.05	0.84174	67.94

## Data Availability

Not applicable.
